# Supplementary material for: Bimodal-Structured 0.9KNbO3-0.1BaTiO3 Solid Solutions with Highly Enhanced Electrocaloric Effect at Room Temperature
Source: Nanomaterials (Basel). 2022 Aug 4;12(15):2674. doi: 10.3390/nano12152674 (PMC9370179; doi:10.3390/nano12152674)
Supplement: Supplementary file 1 [file nanomaterials-12-02674-s001.zip › nanomaterials-1800530-supplementary.pdf]

Bimodal-Structured $0.9KNbO_3$-$0.1BaTiO_3$ Solid Solutions with Highly Enhanced Electrocaloric Effect at Room Temperature

Hongfang Zhang[1,*], Liqiang Liu[2], Ju Gao[3,*], K. W. Kwok[4], Sheng-Guo Lu[5,*], Ling-Bing Kong[6], Biaolin Peng[7], Fang Hou[1]

[1] School of Physical Science and Technology, Suzhou University of Science and Technology, Suzhou, 215009, China
[2] Center for Advanced Ceramics, School of Materials Science and Engineering, Anhui Polytechnic University, Wuhu, 241000, China
[3] School of Optoelectronic Engineering, Zaozhuang University, Zaozhuang, Shandong 277160, China
[4] Department of Applied Physics, The Hong Kong Polytechnic University, Hung Hom Kowloon, Hong Kong
[5] Guangdong Provincial Research Center on Smart Materials and Energy Conversion Devices, Guangdong Provincial Key Laboratory of Functional Soft Condensed Matter, School of Materials and Energy, Guangdong University of Technology, Guangzhou, 51006, China
[6] College of New Materials and New Energies, Shenzhen Technology University, Shenzhen 518118, Guangdong, China
[7] School of Advanced Materials and Nanotechnology, Xidian University, Xi'an 710126, China

***Corresponding authors**: constance_zhanghf@126.com (H. Zhang); jugao@hku.hk (J. Gao); sglu@gdut.edu.cn (S. Lu).

1. Experimental procedure

The preparation procedures of the KN-BT(9/1) compound ceramics with the conventional ceramic processing and sol-gel technique are shown in Figs. S1 (a, b). Fig. S1 (c) shows schematic route of the IAGG method.

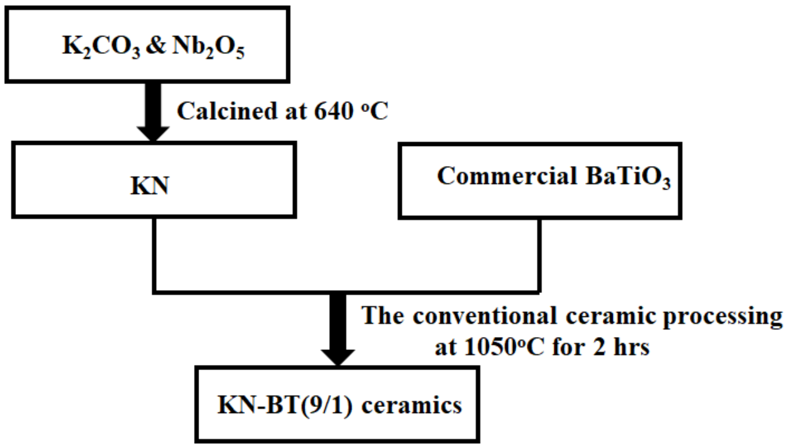

(a) The conventional ceramic processing.

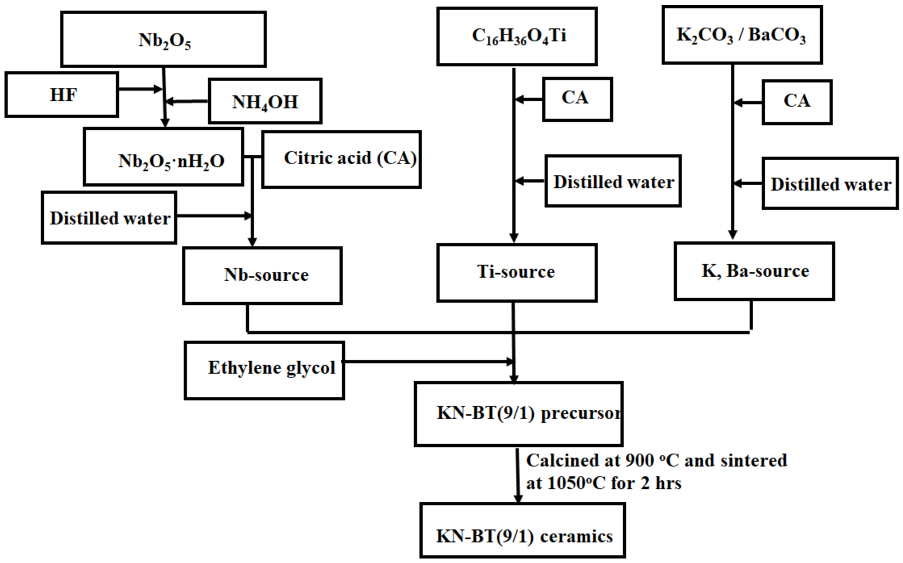

(b) Sol-gel technique using the modified Pechini method.

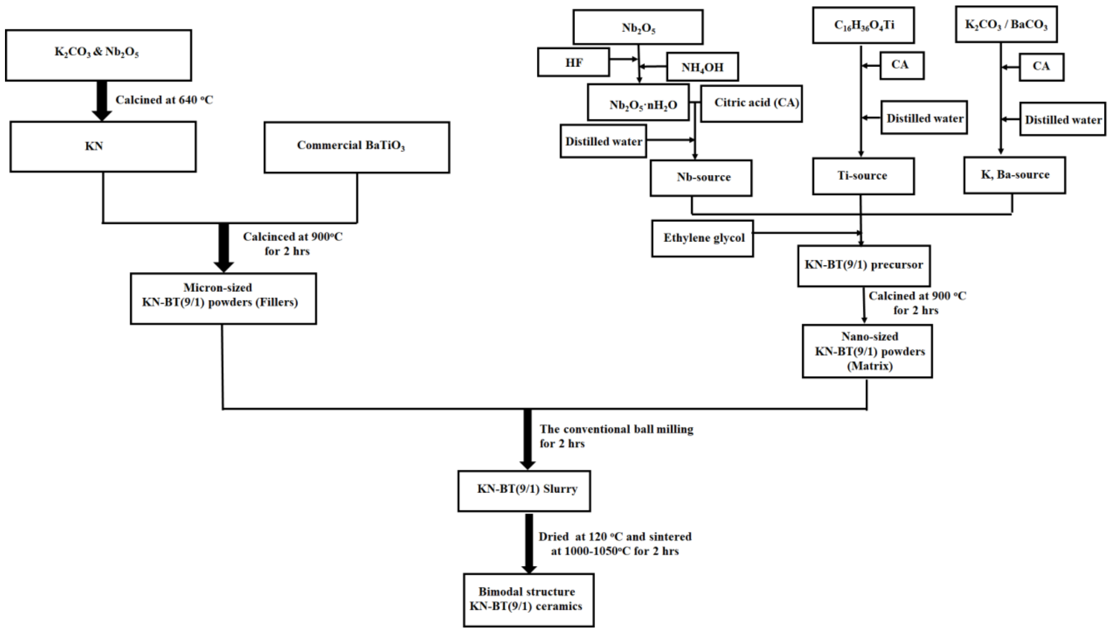

(c) The scheme of an induced abnormal grain growth method (IAGG).

Figure S1. Synthesis routes using (a) the conventional ceramic processing, (b) Sol-gel technique using the modified Pechini method, and (c) The induced abnormal grain growth method (IAGG).

Three processing techniques were used to prepare KN-BT(9/1) ceramics, as shown in Fig. S1, i.e., (a) the conventional ceramic processing, (b) sol-gel technique using the modified Pechini method, and (c) IAGG method. As described in Fig. S1 (c), firstly, the micron-sized KN-BT(9/1) filler powder was dispersed in KN-BT(9/1) sol precursor as the matrix to form uniform suspension. The mass ratio of the filler to the nano-sized KN-BT(9/1) matrix powders derived from the sol precursor at 900 ^{o}C is defined as:

$$W_{fiilers} = \frac{w_{fillers}}{W_{gel\,residue}} \times 100\%, \qquad (1)$$

Where $W_{fiilers}$ and $W_{gel\,residue}$ are the weights of the micron-sized KN-BT(9/1) filler powder (as shown in Figure 2 (b)), and the nano-sized KN-BT(9/1)

matrix powder derived from the sol precursor at 900 ^{o}C (as shown in Figure 2 (c)), respectively. In the present study, $W_{fiilers}$ was confined to 10 wt% by the IAGG method. Then, the homogeneous slurry (suspension) was completely using an agater jar containing agate balls at 200 rpm for 2 hrs with an assistant of ethanol as the milling agent, and then dried at 120 ^{o}C. The dried powder was used to prepare green pellets, all the KN-BT (9/1) green pellets were uniaxially pressed with a diameter of 13 mm and a thickness of about 1 mm at a pressure of 4 MPa in a stainless steel die, and sintered at 1000–1050 ^{o}C for 2 hrs in air with a heating rate of 2 ^{o}C/min.

2. XRD patterns

Fig. S2 (a) shows the XRD pattern of the bimodal structure of KN-BT(9/1) bulk ceramic sintered at 1050 ^{o}C, inset shows the amplified (200) peak at 2 theta (degree): 44–47°. The Rietveld refinement of XRD pattern is shown in Fig. S2 (b) using GSAS refinement software, including Fig. S2 (c) showing the corresponding crystal structure information respectively.

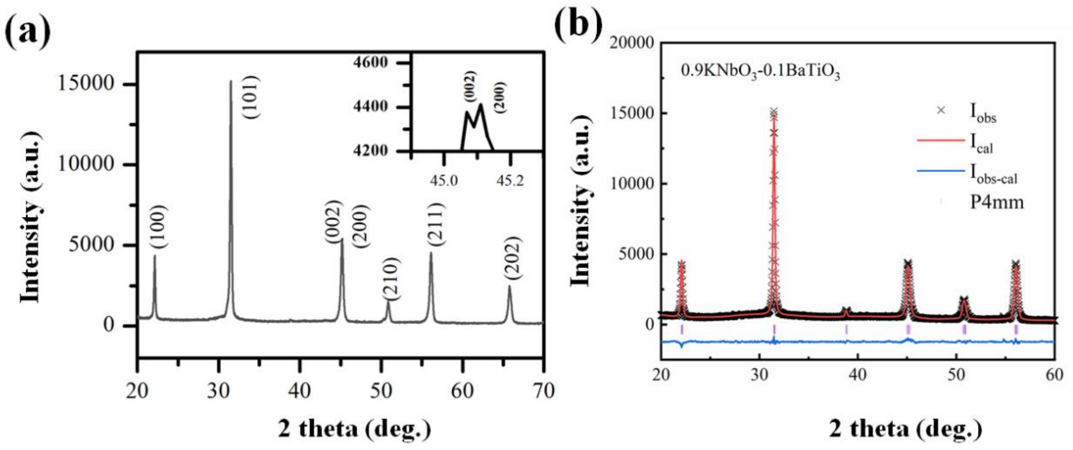

(c) Crystal information using the Rietveld method.

$0.9KNbO_3$-$0.1BaTiO_3$ (Room Temperature)										
Space group	Lattice parameters	Positional coordinates				Occupancy	Uiso	R_{wp}	R_p	χ^2
		Atoms	x	y	z					
		K	0	0	0.0202	0.9	0.0105			
	a=4.012182 Å	Ba	0	0	0.0202	0.1	0.0105			
P4mm	b=4.012182 Å	Nb	0.5	0.5	0.5028	0.9	0.0065	5.10%	3.83%	1.898
	c=4.025965 Å	Ti	0.5	0.5	0.5028	0.1	0.0065			
	V=64.808 $Å^3$	O1	0.5	0.5	0.0205	1	0.0023			
		O2	0.5	0	0.5659	1	0.0023			

Figure S2. (a) XRD patterns of the bimodal structure KN-BT(9/1) bulk ceramics sintered at 1050 ^{o}C using IAGG method. (b) The Rietveld refinement of XRD pattern using the GSAS refinement software, including (c) corresponding crystal structure information through the Rietveld method Inset shows Zoom-in view of 44–47°.

As shown in Figure S(2), the theoretical density value is calculated as 4.7501 g/cm^3. By comparison, Figures S3 shows the Rietveld refinement of the parent XRD patterns, i.e., micron-sized pure $KNbO_3$ powders calcined at 640 ^{o}C, and commercial-grade nano-sized pure $BaTiO_3$ powders, including the corresponding Rietveld refinement information respectively.

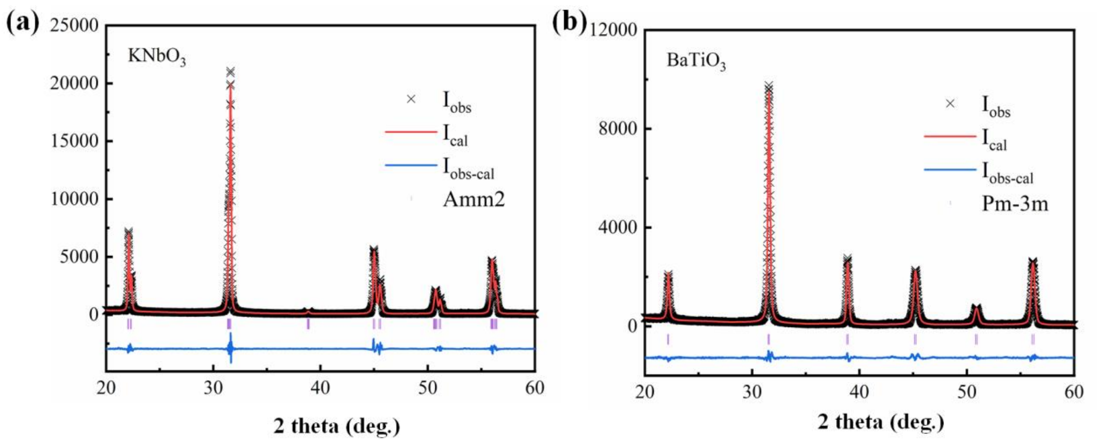

(c) Crystal information of micron-sized pure $KNbO_3$ powders using the Rietveld method.

KNbO3 (Room Temperature)										
**Space group**	**Lattice parameters**	**Positional coordinates**				**Occupancy**	**Uiso**	**R_{wp}**	**R_p**	**χ^2**
		**Atoms**	**x**	**y**	**z**					
Amm2	a=3.986094 Å	K	0	0	-0.0129	1	0.0127	7.71%	5.77%	2.65
	b=5.696096 Å	Nb	0.5	0	0.4579	1	0.0076			
	c=5.706136 Å	O1	0	0	0.6378	1	0.0054			
	V=129.559 $Å^3$	O2	0.5	0.3158	0.2896	1	0.0054			

(d) Crystal information of nano-sized pure $BaTiO_3$ powders using the Rietveld method.

BaTiO3 (Room Temperature)										
**Space group**	**Lattice parameters**	**Positional coordinates**				**Occupancy**	**Uiso**	**R_{wp}**	**R_p**	**χ^2**
		**Atoms**	**x**	**y**	**z**					
Pm-3m	a=4.013290 Å	Ba	0.5	0.5	0.5	1	0.0115	7.78%	5.28%	1.75
	b=4.013290 Å	Ti	0	0	0	1	0.0109			
	c=4.013290 Å									
	V=64.640 $Å^3$	O	0.5	0	0	1	0.0062			

Figure S3. The Rietveld refinement of the parent XRD patterns: (a) micron-sized $KNbO_3$ powders calcined at 640 ^oC, (b) commercial-grade nano-sized $BaTiO_3$ powders. (c, d) Corresponding crystal structure information through the Rietveld method using the GSAS refinement software respectively.

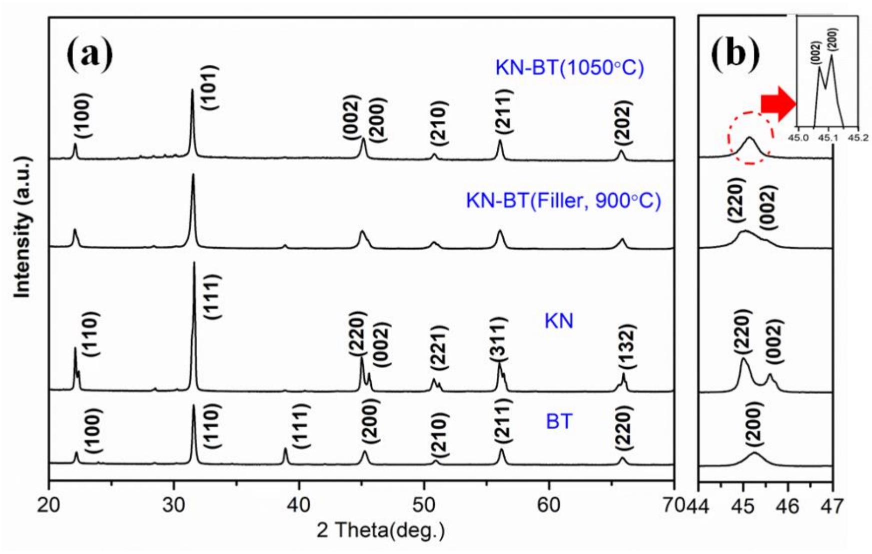

Figure S4. (a) XRD patterns of the commercial nano-sized $BaTiO_3$ (BT) powder, micron-sized $KNbO_3$ (KN) and KN-BT(9/1) filler powders using the conventional ceramic processing, together with a bimodal structure KN-BT(9/1) bulk ceramics sintered at 1050 ^{o}C using IAGG method. (b) Zoom-in view of 44–47°. The dash line and arrow are drawn to guide eyes.

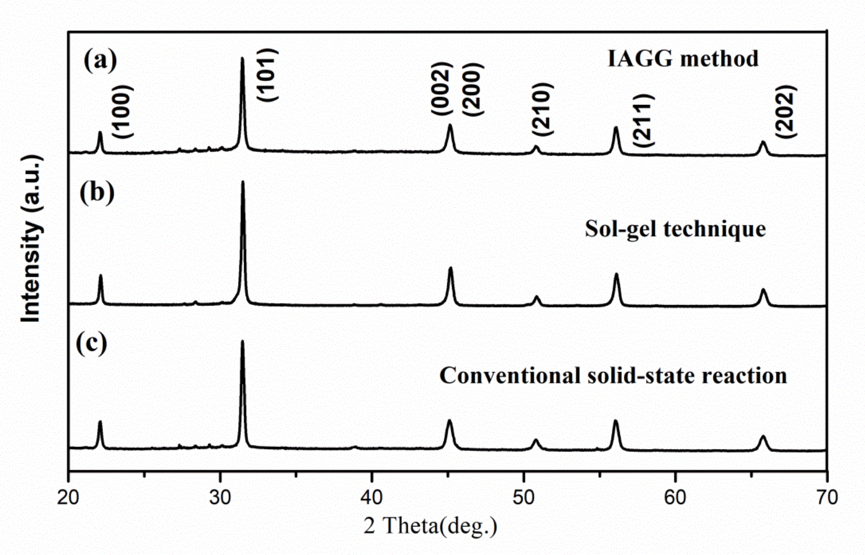

Figure S5. XRD patterns of the KN-BT (9/1) bulk ceramics prepared by using the conventional ceramic processing (i.e., solid-state reaction), sol-gel technique and IAGG method at 1050 ^{o}C respectively.

3. SEM characterization results

(a) Coarse grain

Element	wt%	at%
OK	16.14	45.99
NbL	52.68	25.85
KK	19.87	23.17
BaL	09.32	03.09
TiK	01.99	01.90
Matrix	Correction	ZAF

(b) Fine grain

Element	wt%	at%
OK	16.80	46.88
NbL	54.01	25.95
KK	19.50	22.26
BaL	06.77	02.20
TiK	02.92	02.72
Matrix	Correction	ZAF

Figure S6. The automated element identification for EDS spectra evaluation for (a) Coarse grain. (b) Fine grain respectively.

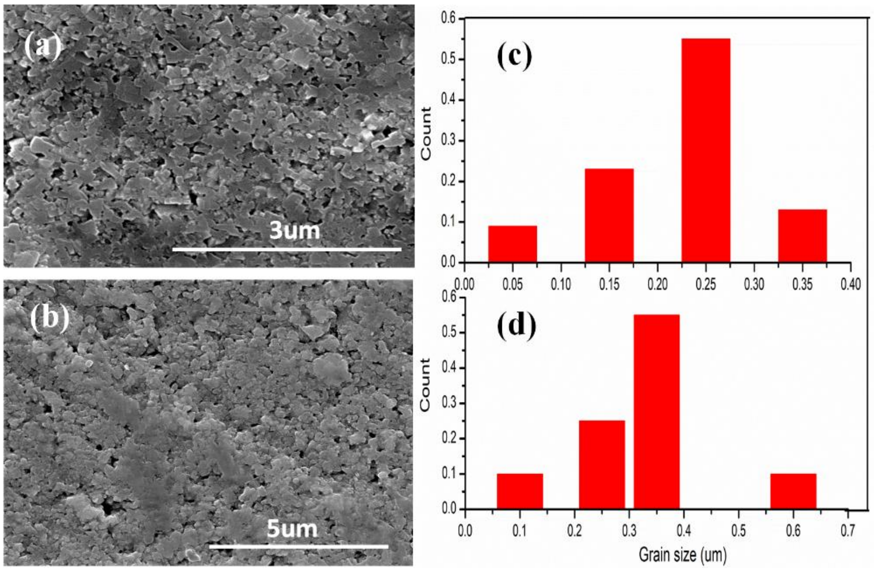

Figure S7. SEM images of the KN-BT(9/1) ceramics prepared at 1050°C by using (a) sol-gel technique and (b) the conventional solid-state processing, (c) and (d) are corresponding to (a) and (b) respectively.

As shown in Fig. S7, the samples showed unimodal structure with the average grain size about 250 nm in Fig.S7(a) and about 300～400 nm in Fig.S7(b) respectively. No abnormal grain growth was observed by the conventional processing, and sol-gel technique.

4. Specific heat capacity

The specific heat capacity and enlarged loss tangent value are shown in Fig. S8.

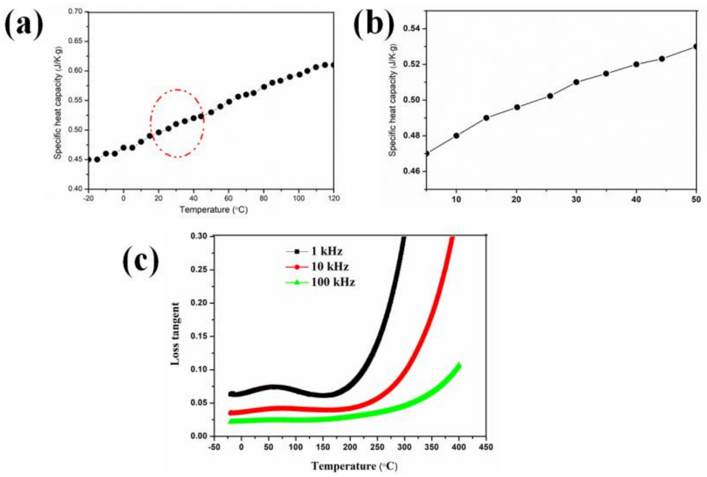

Figure S8. (a, b) The measured specific heat capacity as a function of temperature, together with the enlarge segment between 10–50 °C. (c) The loss tangent dependent of the temperature at different frequency. The red dash circle was drawn to guide eyes.

5. P-E loops

Fig. S9 shows the room-temperature P-E loops of the KN-BT(9/1) ceramics prepared by (a) the conventional ceramic processing, and (b) sol-gel technique at 100 Hz, including the corresponding enlarged segment of P-E loops in (c) and (d) respectively. As shown in Fig. S9(a) and (b), the P-E loops exhibit the obvious lossy loop, compared with IAGG method at the same electric field strength.

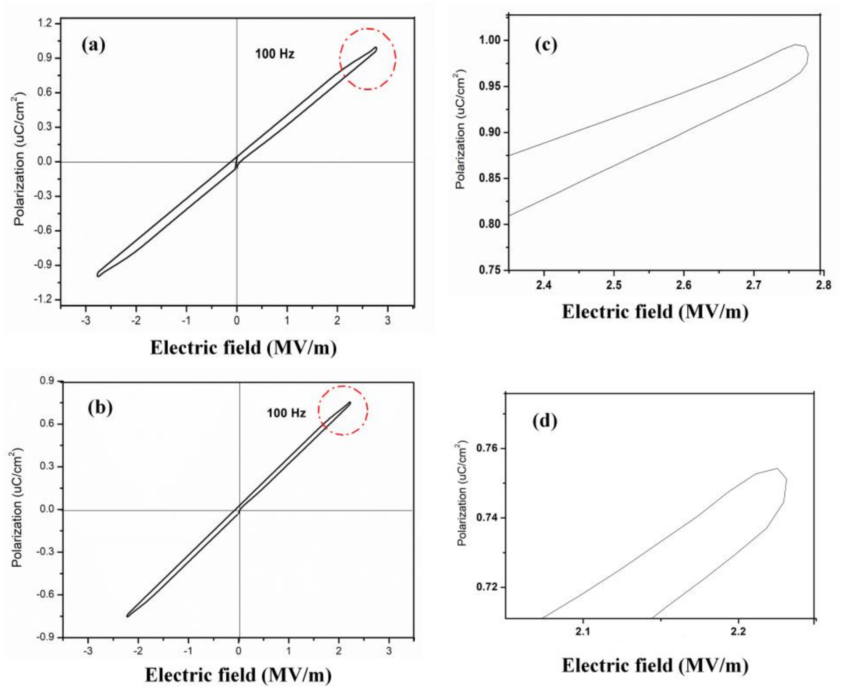

Figure S9. Room-temperature P-E loops of the KN-BT(9/1) ceramics sintered at 1050 ^{o}C using (a) the conventional ceramic processing, (b) the sol-gel technique. The corresponding enlarged segments of loops are shown in the Figs. S9(c) and (d) respectively. The red dash circles were drawn to guide eyes.
